# Editorial: Transitional and long-term continuous care & rehabilitation after stroke

**DOI:** 10.3389/fneur.2022.965762

**Published:** 2022-08-05

**Authors:** Won-Seok Kim, Masahiro Abo, Surjo R. Soekadar, Caterina Pistarini

**Affiliations:** ^1^Department of Rehabilitation Medicine, Seoul National University College of Medicine, Seoul National University Bundang Hospital, Seongnam, South Korea; ^2^Department of Rehabilitation Medicine, Jikei University School of Medicine, Tokyo, Japan; ^3^Clinical Neurotechnology Lab, Department of Psychiatry and Neurosciences, Charité–Universitätsmedizin Berlin, Berlin, Germany; ^4^Department of Neurorehabilitation Istituti Clinici Scientifici Maugeri, Istituto di Ricovero e Cura a Carattete Scientifico (IRCCS), Pavia, Italy

**Keywords:** stroke, rehabilitation, transitional care, long-term care, clinical trial, protocol, observational study

Stroke incidence and prevalence are increasing, causing substantial socioeconomic burdens and loss of healthy life-years worldwide (1). Timely acute treatment, such as thrombolysis, is crucial to reduce mortality and disability; however, many stroke survivors have to live with disabilities after acute care (1–4). Therefore, continuous rehabilitation and long-term care to meet the needs of individual stroke survivors is mandatory.

Establishing novel therapeutics to promote the recovery and management to avoid complications along with adequate rehabilitation in acute and subacute phases is important because the time window for recovery after stroke is limited (5). After adequate intensive rehabilitation, many stroke survivors and their caregivers lack a personalized long-term continuous care plan. Moreover, important information has often not been provided at the time of discharge from acute treatment. Because such need for long-term care is unmet in many cases, subjective and objective outcomes remain unfavorable (6). Therefore, adequate transitional and long-term care strategies and therapeutics to compensate or reduce the effect of impairments after stroke are required.

The Research Topic “Transitional and Long-term Continuous Care & Rehabilitation After Stroke” included 14 manuscripts, ranging from a study protocol, case report and observational study to randomized controlled trials, a mini-review, systematic review, and meta-analysis. These timely contributions offer an interesting insight from acute rehabilitation to transitional and long-term care in patients with stroke.

Effective rehabilitation and medical management to promote functional recovery and reduce complications are important, and three related papers in this area were presented. A large randomized controlled trial (AVERT) showed that early mobilization within 24 h of stroke onset was feasible but not effective in terms of favorable outcomes defined as a modified Rankin Scale (mRS) score of 0–2 at 3 months post-stroke (7). However, the prespecified dose–response analysis from the AVERT revealed that shorter and more frequent mobilization early after acute stroke can be beneficial (8). One randomized controlled trial on intracerebral hemorrhage demonstrated that early rehabilitation within 48 h can improve functional outcomes and reduce mortality (9). A recent randomized controlled trial in patients with mild and moderate intracerebral hemorrhage also revealed that early mobilization within 24 to 72 h of stroke onset can improve the early functional independence (10). Therefore, the study aimed to investigate the effect of early mobilization after stroke in various stroke patients' group in terms of stroke type or severity and using different mobilization protocol in terms of timing, frequency, session duration is needed. Wang et al. conducted a randomized controlled trial investigating the effect of early out-of-bed mobilization within 48 h of stroke symptom onset compared to the conventional rehabilitation group in the unique patient group who underwent mechanical thrombectomy. In this study, early mobilization reduced non-fatal complications 3 months post-stroke and improved the activities of daily living at 3 months and 1-year post-stroke, although the effects on mortality and mRS were comparable with that of conventional rehabilitation.

Dysphagia is a common impairment after stroke that is considered important due to its association with the incidence of pneumonia as confirmed by Chang M. et al. (OR 9.60; 95% CI 5.75–16.05) in their systematic review and meta-analysis of five studies.

Statins have a potential to promote recovery after stroke by their possible pleiotropic effects; however, their results in human studies are controversial (11–13). Mele et al. retrospectively analyzed the data of 413 patients with stroke from a single neurorehabilitation center and reported that statin therapy was not associated with recovery and functional outcomes but associated with a lower incidence of bone fractures.

Earlier subacute stroke rehabilitation trials are necessary, considering the preclinical studies reporting that earlier rehabilitation after stroke is better for neuromotor recovery (14, 15). However, previous stroke rehabilitation trials have conventionally focused on patients at >3–6 months post-stroke. Geed et al. reported screening and enrollment data from the Critical Periods After Stroke Study (CPASS), which recruited patients within 30 days of stroke (16). In an acute care setting, a short length of stay and prior stroke were the major reasons for exclusion in CPASS. In an inpatient rehabilitation setting, “too late” to participate in an early stroke trial, prior stroke and too mild impairment were the major reasons for exclusion. The Mean Action Research Arm Test score for the affected upper extremity in a patient with mild impairment (NIH stroke scale for motor arm item = 0 or 1) was 39 (max score = 57), which indicates significant impairment having a potential benefit from participation in upper limb rehabilitation trials. Therefore, patients with stroke should be screened when they are in the acute care phase, and screening of patients with mild impairment should be conducted using a motor function specific scale for successful recruitment for early stroke rehabilitation trials.

Our topic includes four manuscripts regarding the issues in transitional care strategies or systems after stroke. One of the transitional care strategies after stroke is “early supported discharge (ESD)” aiming to expedite home discharge after stroke by providing adequate management and rehabilitation services after discharge in the patient's home. ESD has been known to reduce long-term dependency and length of the hospital stay; however, most studies on ESD have been conducted in western countries (17). Moreover, the recent meta-analysis (18 studies from western countries and two studies from eastern countries) by Jee et al. reported that ESD and transitional care in patients with stroke did not have significant beneficial effects on functional outcomes, mortality, caregiver strain, and length of hospital stay. ESD or transitional care services should be implemented in accordance with each specific national medical rehabilitation pathways. Chang W. et al. proposed the study protocol for a multicenter randomized controlled trial to investigate the effect of ESD compared with the conventional rehabilitation on activities of daily living, patient- or caregiver-reported outcomes, and socioeconomic outcomes in 90 Korean patients with post-acute stroke with mild-to-moderate disability. Kinoshita et al. and Leigh et al. elaborated on the transitional and long-term care system in Japan and South Korea, respectively, which will be helpful for making comparisons to improve long-term stroke care.

Stroke survivors can have long-term stroke-related impairments or complications associated with poor quality of life. Kim et al. conducted a face-to-face cross-sectional survey to investigate the unmet needs for rehabilitative management in common health-related problems after stroke. Approximately half of the respondents reported at least one unmet need, and the total number of unmet needs was significantly associated with a poor health-related quality of life after adjusting for age, sex, and mRS.

Post-stroke spasticity is associated with complications such as contractures pressure ulcers or pain and poor quality of life (18, 19). Adequate treatments for spasticity can improve patient function and quality of life (20, 21). Zhang et al. conducted a multicenter randomized controlled trial to investigate the effect of dry needling at the myofascial trigger point (30 min in each session for a total of five sessions for 4 weeks) on hand spasticity in 210 patients with chronic stroke. The dry needling demonstrated significantly better immediate spasticity relief than sham needling and control groups, and the spasticity relief persisted at 4 weeks after baseline. Fan et al. presented a randomized, double-blind, sham-controlled trial protocol to investigate the short-term effects of radial extracorporeal shock wave therapy on flexor spasticity of the upper limb in 100 patients with >1 month first-ever stroke and elbow joint spasticity grade >1, determined using the modified Ashworth scale.

The incidence of sarcopenia in patients with stroke ranges from 14 to 54% and is associated with poor outcomes (22). Park et al. prospectively enrolled patients with <3 months after stroke, who ingested dietary supplement power containing 6 g of branched-chain amino acids twice a day for 1 month. They selected the control group by a retrospective medical chart review to balance the age and stroke lesions with the intervention group. The branched-chain amino acid supplementation group demonstrated better improvement in skeletal muscle index measured by dual-energy X-ray absorptiometry and in functional scales such as the modified Barthel index.

Novel therapeutics for stroke motor recovery or compensating the impaired function are needed especially for patients with more severe impairments considering their poor recovery potentials (23). Singh et al. reported a case of a 52-year-old woman with a 9-year chronic stroke, who received 20 therapy sessions (45 min/session) with robotic exoskeleton assistance to extend the wrist and flex the fingers in the affected upper limb triggered by electromyography activity of the extensor digitorum communis. She demonstrated improvements in the Fugl–Meyer Assessment score, Barthel index, and modified Ashworth scale with relevant changes in neurophysiologic parameters. Finally, Angerhöfer et al. reviewed the need for brain–computer interface (BCI)-enabled neurorehabilitation in severe upper limb paresis. Specifically, the authors discussed how BCI-based long-term treatment strategies could be established in Germany and outlined the challenges in this process. This review will be an excellent reference for researchers who are interested in effective BCI applications and their clinical implementation.

## Author contributions

All authors listed have made a substantial, direct, and intellectual contribution to the work and approved it for publication.

## Conflict of interest

The authors declare that the research was conducted in the absence of any commercial or financial relationships that could be construed as a potential conflict of interest. The handling editor TP declared a past collaboration with the author(s) W-SK and CP.

## Publisher's note

All claims expressed in this article are solely those of the authors and do not necessarily represent those of their affiliated organizations, or those of the publisher, the editors and the reviewers. Any product that may be evaluated in this article, or claim that may be made by its manufacturer, is not guaranteed or endorsed by the publisher.

## Abbreviations

mRS: modified Rankin scale
CPASS: critical periods after stroke study
ESD: early supported discharge
BCI: brain–computer interface.
